# *Aspergillus* Genus and Its Various Human Superficial and Cutaneous Features

**DOI:** 10.3390/pathogens10060643

**Published:** 2021-05-23

**Authors:** Yassine Merad, Hichem Derrar, Zoubir Belmokhtar, Malika Belkacemi

**Affiliations:** 1 Department Parasitology-Mycology, ‘Hassani Abdelkader’ Hospital, UDL Faculty of Medicine, Laboratoire de Synthèse de L’information Environementale, UDL, Sidi-Bel-Abbes 22000, Algeria; 2 Department of Pulmonary Diseases, ‘Hassani Abdelkader’ Hospital, UDL Faculty of Medicine, Sidi-Bel-Abbes 22000, Algeria; hichem_derrar@yahoo.fr; 3 Department of Environmental Sciences, Faculty of Natural Science and Life, University Djilali Liabes, Sidi-Bel-Abbes 22000, Algeria; zoubir_31@yahoo.fr; 4 Department of Hemobiology and Blood Transfusion, ‘Hassani Abdelkader’ Hospital, UDL Faculty of Medecine, Sidi-Bel-Abbes 22000, Algeria; belkacemi_malika@yahoo.fr

**Keywords:** *Aspergillus*, cutaneous, burns, trauma, otomycosis, onychomycosis

## Abstract

Superficial and cutaneous aspergillosis is a rare fungal disease that is restricted to the outer layers of the skin, nails, and the outer auditory canal, infrequently invading the deeper tissue and viscera, particularly in immunocompromised patients. These mycoses are acquired through two main routes: direct traumatic inoculation or inhalation of airborne fungal spores into paranasal sinuses and lungs. Lesions are classified into three categories: otomycosis, onychomycosis, and cutaneous aspergillosis. Superficial and cutaneous aspergillosis occurs less frequently and therefore remains poorly characterized; it usually involves sites of superficial trauma—namely, at or near intravenous entry catheter site, at the point of traumatic inoculation (orthopaedic inoculation, ear-self-cleaning, schizophrenic ear self-injuries), at surgery incision, and at the site of contact with occlusive dressings, especially in burn patients. Onychomycosis and otomycosis are more seen in immunocompetent patients, while cutaneous aspergillosis is widely described among the immunocompromised individuals. This paper is a review of related literature.

## 1. Introduction

*Aspergillus* species are a large group of common saprophytic moulds which are isolated from soil, air, and plant materials. *Aspergillus* species can cause a group of superficial and cutaneous mycoses: distal lateral subungual onychomycosis, proximal subungual onychomycosis, otomycosis, and cutaneous aspergillosis [1,2].

Almost all *Aspergillus* skin infections are nosocomially acquired, particularly in newborn or immunocompromised patients or following medical procedures such as surgery, catheter insertion, or after occlusive dressings in burn patients [3,4,5]. On the other hand, cutaneous trauma and injuries are also the main risk factors of *Aspergillus* superficial and cutaneous features [6,7,8].

Lesions are commonly solitary and typically develop on the catheter site of insertion, wound dressing, and sites of trauma. The lesions arise as papules, nodules, or ulcerations, or they can mimic dermatophytosis [2,9]. 

## 2. Source of Infection 

The initial step of aspergillosis is colonization of the sites, such as the auditory canal, wounds, injuries, and fragile nails. The origin of infection may be conidia of the fungus from the surrounding air that fell and contaminated a wound [10]; conidia can also be inoculated into skin after injury or surgical procedures. 

*Aspergillus* airborne organisms may be introduced into the body (ear, wound, skin) by an object or following a post-operative procedure; otomycosis may also be considered as a super-infection following a bacterial otitis [11,12,13,14].

## 3. Risk Factors

### 3.1. Environmental Factors 

#### 3.1.1. Outdoor Conditions 

*Aspergillus* species are ubiquitous environmental moulds present in air, soil, water, and decaying vegetation [10,15,16]. Beany and Broughton have attributed the greater frequency of otomycosis in the tropical countries to changes in the composition of cerumen induced by sweating; furthermore, seasonal variations that have been reported in the incidence of filamentous fungal mycosis have been related to many environmental factors, such humidity, wind, and rainfall and have been also linked to the harvest [16,17,18]. 

#### 3.1.2. Hospital Conditions 

Because catheter insertions and surgeries are typically done in hospital, almost all skin aspergilloses are hospital-acquired infections; moreover, umbilical catheter infection by *Aspergillus* species have been reported in newborns, and *Aspergillus* wound infection is recognized among burn patients treated in hospital [3,4,6].

Malfunctions in healthcare facility systems, improper installation, filter damage, and poor maintenance can facilitate the spread of healthcare–associated airborne infections [19].

### 3.2. Host Factor

**Figure 1:**
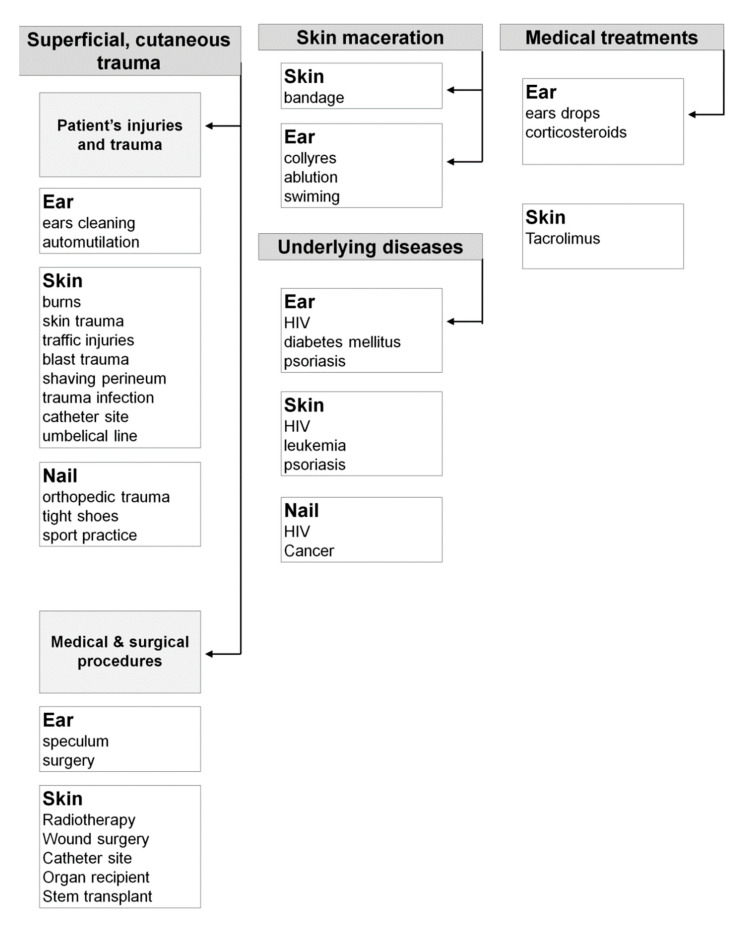
Etiological factors of superficial and cutaneous aspergillosis.

The outcome of aspergillosis depends more on host factors than on the virulence of the *Aspergillus* species [20,21,22] (Figure 1).

Onychomycosis is more common in the elderly [23,24,25]. Premature infants and newborns are at increased risk of developing superficial and cutaneous aspergillosis; it is widely maintained that the immature immune system of the preterm infant, along with vulnerable skin barrier function, are major factors for superficial and cutaneous aspergillosis [26,27,28,29]. 

Poor hygiene, barefoot walking, sweating, and paronychia predispose to onychomycosis; moreover, occupational exposure such as household chores and gardening can provoke aspergillosis [18], which is more common in immunosuppressed patients [23,24,25].

#### 3.2.1. Physical Activities and Occupational Exposure 

Onychomycosis, induced by *Aspergillus* genus, occurs more commonly than realized, especially in outdoor workers and in agricultural communities [12]. Several patients reported dystrophic nail abnormalities or nail trauma prior to the onset of the mould onychomycosis [30].

Onychomycosis is associated with barefoot walking, and paronychia predispose to onychomycosis, especially in sport activities (athlete’s foot) [18]. *Aspergillus* onychomycosis is seen more among individuals with occupational exposure, as described among vegetable vendors and babassu coconut breakers [31,32,33] and among patients after exposures such as gardening or household chores [18]. Many reports have depicted primary infection in immunocompetent patient in association with previous agricultural trauma [34].

Otomycosis caused by filamentous fungi is usually seen among communities of fisherman [17]. Swimming and other water activities are important because many people swim and dive. Sometimes the external auditory canal is directly exposed to water without any protection [17,35,36].

#### 3.2.2. Local Humidity and Skin Maceration 

Various factors have been suggested as predisposing factors for otomycosis, including swimming regularly and living in a humid environment [17]. One of the contributing factors to this condition is the removal of the protective coating of cerumen by repeated washing and cleaning of the ear canal. Moreover, the right diagnosis and treatment of otomycosis needs a high degree of suspicion in refractory cases of otorrhea; it is clear that both trauma and excessive moisture impair the ear’s natural defences [17,37]. High humidity provides satisfactory conditions for fungal growth [38]. Kim et al. described a case of *Aspergillus* onychomycosis: the patient used to plant beans and therefore her hands were generally exposed to water for long periods of time. Thus, occupation seems be closely linked to the disease’s development [30], especially when one is in continuing contact with water, detergents, and chemical products. 

Furthermore, some religious, cultural, or aesthetical practices leave the external ear canal wet (ablutions, wearing a scarf or veil) [39], which may predispose people to otomycosis induced by *Aspergillus* genus. 

#### 3.2.3. Trauma Exposure 

*Aspergillus* conidia can develop and grow on damaged skin. Usually, patients have been exposed to several kind of trauma.

##### Ear Trauma 

Some traumas are induced by the patient’s habits, such as ear self-cleaning leading to otomycosis. Moreover, self-inducted injuries are already described in schizophrenia, and *Aspergillus flavus* otomycosis has been linked to ear self-mutilation [7].

##### Nail Trauma

Athlete’s foot is induced by regular sport activities, which is well described in literature [23,30]; even the habit of wearing tight shoes can induce toenails onychomycosis [40].

##### Skin Trauma

Primary cutaneous aspergillosis has been related to agricultural trauma, orthopaedic trauma [1,34], or traffic injuries [41]; usually, the symptoms appear within one month of injury [41].

Panke et al. [42] observed a “fruiting bodies” of *Aspergillus* on the skin of a burned patient. Furthermore, burn wounds can be infected by *Aspergillus* genus [6,43,44].

A primary cutaneous aspergillosis may act as a source of fungal dissemination to various organs of the body, including the lungs, the heart, and the central nervous system [13].

Septic shock caused by *Aspergillus fumigatus* infection was reported in a patient with a lacerated lower limb after being injured in a factory blast; the cause of septic shock was the existence of the fungi within the wound [45].

A 31-year-old healthy female presented with multiple axillary and perineal ulcers following incision and drainage of slowly growing nodular lesions over a one-year duration. She admitted to shaving her axillae and pubic region with a safety razor several times during the one-year period. Culture and histology revealed a significant growth of *Aspergillus* [8].

Some of the *Aspergillus* infections induced by trauma are summarized in Table 1.

#### 3.2.4. Underlying Medical Conditions

Usually, aspergillosis starts from a lung infection subsequent to inhalation of airborne spores. Moreover, in the immunocompromised patient, hematogenous dissemination and invasion of other organ systems, including skin, often follows primary pulmonary infection. Cutaneous aspergillosis can be considered as a cutaneous manifestation of disseminated infection with the *Aspergillus* species.

Primary cutaneous aspergillosis was previously described in immunocompromised patients [49,50], especially in HIV patients [2,4,48,49,50,51,52].

Shetty et al. [4] described a cutaneous non-healing ulcer on the right calf muscle of an HIV infected child, with no wound dressing and no previous trauma. The skin biopsy specimen revealed hyphal elements, and the culture of the sample grew *Aspergillus glaucus.*

Several reports have described primary or secondary cutaneous aspergillosis in immunocompromised patients who are not infected with HIV, including burn victims, neonates, cancer patients, solid-organ and bone marrow transplant recipients [53,54,55,56,57,58,59].

Diabetes mellitus and hyperglycaemia from hyper-alimentation are additional risk factors in burn patients [51]. Diabetic burn patients seem to have a higher rate of infection in comparison with non-diabetic burn patients [60].

In a literature review, *Aspergillus niger* skin infection following bone marrow transplant was also described [58], as well as cutaneous aspergillosis following kidney transplant [60,61]. Moreover, multiple *A. fumigatus* inflammatory nodules of the lower limb were reported after a liver transplant [12].

Furthermore, unique painful, necrotic nodule of the calf muscle, toe paronychia, and catheter site necrosis were induced by *Aspergillus flavus* in leukaemia patients [12], and invasive aspergillosis can occur in the course of cutaneous aspergillosis-associated with acute myeloid leukaemia [62].

On the other hand, diabetes mellitus has not been identified as a risk factor for invasive aspergillosis in the general population. However, in the *Aspergillus* infections literature, the proportion of patients with diabetes mellitus tended to be high [63]. Diabetes mellitus has also been present in relation with *Aspergillus* nails infections. The latter is an emerging onychomycosis pathogen among diabetics, and the risk of having *Aspergillus* nail disorders among patients being treated for diabetes increases with the duration of the disease [64]. In a study conducted in India among patients who were involved in agricultural activities, 77% were diabetic and were confirmed to have *Aspergillus* onychomycosis [64]. Nail damage can be noticed in HIV patients and in patients with hormonal imbalance induced by Cushing’s syndrome and hypothyroidism.

Some additional underlying diseases have been associated with cutaneous aspergillosis; they are summarized in Table 2.

#### 3.2.5. Medical Procedures

Different medical procedures are related to superficial and cutaneous *Aspergillus* infections, such as exploratory medical procedures, medical treatments, and surgery.

Smith and Wallace reported a cutaneous lesion of a patient under radiation therapy for non-Hodgkin’s lymphoma; the lesion was located under the dressing of a venous catheter, and histopathology revealed numerous branching hyphae within the follicular infundibulum [69]. Total parenteral nutrition in burn patients is a risk factor of fungal infection [51,70].

##### Medical Devices

Predisposing factors of *Aspergillus* otomycosis include the use of hearing aids with occlusive ear mould that may provoke accumulation of cerumen and epithelial debris in the external auditory canal [71]. The catheters are widely used, and they can be the most common source of *Aspergillus* infection.

##### Insertion of Catheters

Many of the cutaneous aspergillosis infections in the early 1980s and 1990s were related to Hickman catheters [2,48]. The initial mechanism of aspergillosis involves making a tunnel through the skin, allowing for a direct inoculation of fungi [2,46,49]. In cutaneous Aspergillosis, the lesions usually develop at the site of catheterization [72]; they can be erythematous and indurated [48].

Greenish lesions in the umbilical region of two preterm twins were described. The catheters were removed, and the culture was positive for *Aspergillus fumigatus* [73]. Central catheters are currently used in burn patients, especially in severe cases with prolonged IUC stay [70].

##### Applying Bandages, Dressings and Gauze

In some cutaneous Aspergillosis, the lesions are generally located at points of contact with gauze or dressings [26]. Smith and Wallace reported a cutaneous lesion under the transparent dressing of a venous catheter [69].

Bandages may be a source of infection [72]. Burn wound dressings are carried out by either the open or occlusive method. Open dressings are associated with a higher incidence of infection than occlusive dressings [10], as are those with extensive burns (>50%).

Recently, primary cutaneous aspergillosis with *Aspergillus niger* at the place of skin abrasion that had been managed by a cyanoacrylate topical skin adhesive was described [74].

##### Medical Instrumentation

In otomycosis, predisposing factors include ear medical instrumentation [14,71]. Ozer et al. described a case of cutaneous infection caused by *Aspergillus terreus* in a paediatric patient who underwent surgical treatment for an open tibial fracture [34].

Anderson et al. reported an ear surgical wound healing that was complicated by *Aspergillus flavus* infection in a non-immunocompromised patient.

Primary cutaneous aspergillosis can occur directly in the surgical wound among liver or renal transplantation patients [13,61,66].

##### Medical Drugs

Fungal infections are a well-known complication of broad-spectrum antibacterial use. Furthermore, otitis may be exacerbated by the prescription of broad-spectrum antibiotics such as fluoroquinolone eardrops [75].

Currently, mixed bacterial and fungal otitis are the consequence of long courses of bacterial otitis treatment, leading to the alteration of the normal ear flora [14].

Corticosteroids significantly impair the functionality of innate immunity, and steroid-induced hyperglycaemia might further weaken the innate immune response against mould infections such as Aspergillosis [63,70].

Agranulocytosis treated with antithymocyte globulin was related to skin aspergillosis [50,51]

Prolonged hospital stay and the use of broad spectrum antibiotics were related to cutaneous *Aspergillus* in burn patients [10], and the incidence may have risen due to the suppression of bacterial infections with use of silver sulphadiazine and prompt surgical excision [70].

## 4. Cutaneous and Superficial Aspergillosis

### 4.1. Otomycosis

Otomycosis is a fungal superficial, subacute or chronic infection of the external auditory canal with some rare invasive complications involving the middle and inner ear. Pruritus is the most common fungal symptom of otomycosis [38]. Rarely, otomycosis can spread to nearby structures, such as eardrum, bone, and cartilage, particularly in immunocompromised patients, and especially by *Aspergillus* species [76]. In addition, *Aspergillus niger* was seen in chronic unilateral otomycosis and invasive otitis externa [77]. *Aspergillus* otomycosis is relatively frequent. Mycological study of otomycosis in the eastern part of Maharashtra (India) revealed 25% of *Aspergillus* cases [78].

**Figure 2:**
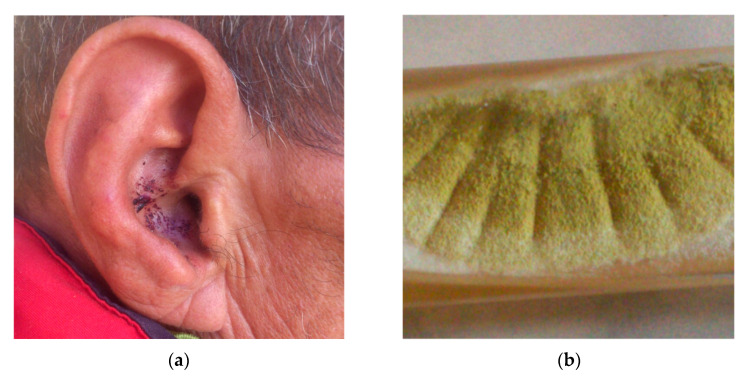
(**a**) Clinical appearance of self-induced otomycosis in a schizophrenic patient; (**b**) macroscopic aspect of *Aspergillus flavus.*

Predisposing factors include living in a humid climate, moisture, bathing, the presence of excessive cerumen, wearing turbans, repeated use of topical antibiotics and steroids or ear oil instillation [17,35,36], medical instrumentation, self-cleaning of the ear with foreign or unsterilized objects, and ear auto-mutilation in schizophrenia (Figure 2). Furthermore, *Aspergillus* otomycosis can be seen in immunocompromised hosts, after open-cavity mastoidectomy surgery, after the accumulation of epithelial debris in the external auditory canal, and after the use of hearing aids with an occlusive ear mould [36,78]. Many factors encourage infection and changes in the epithelial covering. High humidity creates perfect conditions for fungi growth [38]. The other predisposing factors are dermatological diseases such as dermatophytosis, the loss of cerumen, and the use of topical broad-spectrum antibiotics [14].

The predominant fungal pathogens in otomycosis are different in various literature reports (Table 3). They include *Aspergillus flavus* [79] (Figure 2), *Aspergillus niger, Aspergillus fumigatus, Aspergillus versicolor, Aspergillus candidus*, and *Aspergillus persii* [17].

In a study conducted in Egypt, the majority of fungal otitis cases were related to *Aspergillus* (84.8% of cases) [80]; furthermore, *Aspergillus* is considered to be the most prevalent otomycosis agent in India and China [81,82].

In chronic otitis media, different isolates of *Aspergillus* species are described (*Aspergillus niger, Aspergillus flavus, Aspergillus fumigatus, Aspergillus terreus*) [83,84]. Most patients suffer from complaints of pruritus, otorrhea, otalgia, tinnitus, and blocking sensation [85,86].

Otoscopy may reveal variable black, green, or grey fluffy elements in the ear canal when *Aspergillus* is present.

It is not uncommon for otomycosis to develop in patients following acute bacterial otitis media with otorrhea [71] or with the auditory canal showing oedema, erythema, or exfoliation of the epithelium [12].

Since clinical features of otitis are not specific, laboratory diagnosis is essential to define the correct aetiology of otomycosis and to identify effective antifungal therapy, depending on the type of otitis and the fungal pathogen. Certain otomycoses may reveal microscopic images that are highly suggestive of the etiological agent. Microscopy typically shows numerous *Aspergillus* heads and abundant septate hyphae, in addition to multiple specific fungal structures including microconidia.

### 4.2. Onychomycosis

Onychomycosis, known as tinea unguium, is a chronic fungal infection of the toenails or fingernails that is usually not painful but can affect a patient’s quality of life by interfering with footwear. The toenails are more frequently involved than fingernails. Onychomycosis may affect up to 30% of the population by age 60 and 70.

**Figure 3:**
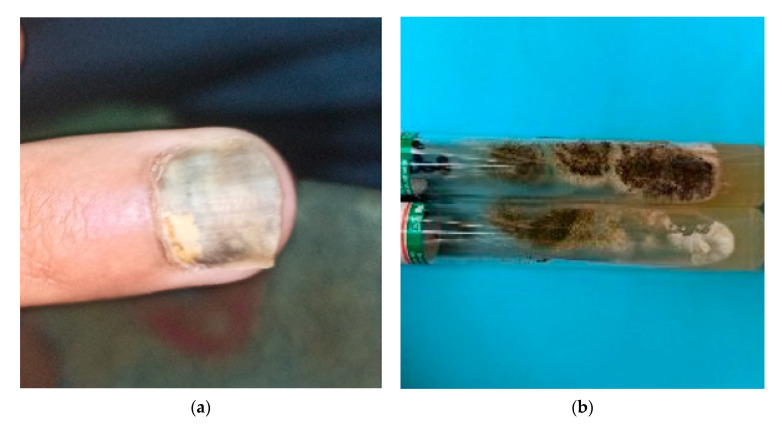
(**a**) *Aspergillus* onychomycosis induced by trauma in a vegetable vendor patient; (**b**) *Aspergillus flavus* culture macroscopy.

It is mostly caused by dermatophytes, and particularly by *Trichophyton rubrum* [96]. *Aspergillus* species are the second most frequent agents of non-dermatophytic onychomycosis [97]. Onychomycosis due to *Aspergillus* species is rare (Figure 3) (Table 4), ranging from 2% to 30% of all cases, and the prevalence is higher among diabetic patients, accounting for almost 71%. [1,15,97].

In addition, it is speculated that *Aspergillus* strains can have a clear keratinophilic activity, which causes partial or total dystrophy of the affected nails [98]. A review of the relevant literature has shown that there are at least 11 species of *Aspergillus* which have been found in onychomycoses, either alone or in association with other known pathogens. Such cases usually occur as a result of trauma or colonization [34]. In Malaysia, according to Leelavathi et al. *Aspergillus sp.* was the main fungus isolated in onychomycosis (59,8%, *n* = 71), and the mixed cultures of *Aspergillus* accounted for almost 15,1%. The latter was in combination with fungi such as *Penicillium* or other non-dermatophytes fungi [99].

Diabetes, peripheral vascular disease, orthopaedic trauma, and advanced age are the most important underlying conditions in onychomycosis due to *Aspergillus* species, although no risk factors are evident in most of cases [1].

Numerous non-dermatophyte filamentous fungi are usually isolated as commensals from damaged nails, mostly from the toenails of elderly [100]. The toenails are involved more frequently than fingernails due to important exposure to soil, water, and decaying vegetation where *Aspergillus* moulds flourish [32].

Toenails are more affected by onychomycosis than fingernails, which is probably because of their slow growth and is perhaps encouraged by external factors such as trauma and poor circulation [101].

Dhib et al. included 7151 patients (4709 women and 2442 men) with clinical suspicion of onychomycosis; moulds accounted for 4.2% of cases, and *Aspergillus sp* was the most frequent one [102].

The clinical characteristics suggesting onychomycosis due to *Aspergillus sp* are chalky white nail, rapid involvement of lamina, and painful perionyxis without pus [97]. Kara et al. reported onychomycosis due to *Aspergillus flavus* involving all fingernails and toenails of an immunocompromised patient [103]. In rare cases, physical examination can reveal pronounced dystrophy of the nail plate, onychoclasis, and onychomadesis with black discoloration of the proximal nail bed [100].

To definitely set up a diagnosis of aspergillosis of the nails, one should not depend fully on the clinical signs, which by themselves may be confusing.

### 4.3. Cutaneous aspergillosis

Cutaneous diseases of multiples aetiologies are commonly encountered in human clinical practice; cutaneous aspergillosis is usually a skin presentation of disseminated infection with the genus *Aspergillus.* Initial cutaneous disease is infrequent and is most commonly caused by *Aspergillus fumigatus*, *Aspergillus flavus*, *Aspergillus terreus,* and *Aspergillus ustus*.

Primary cutaneous aspergillosis occurs in the sites of direct skin lesion or injury following surgery, burns, trauma, occlusive dressing, or intravenous cannulation [3,26]. It can also happen directly in the surgical wound among renal or liver transplantation patients [13,61,66]. On the other hand, primary cutaneous aspergillosis should be suspected in the significantly low birth weight population with fast progressive ulcerating and necrotic skin lesions [3].

Secondary cutaneous aspergillosis spreads through hematogenous dissemination to the skin from a distant point. Usually, it follows the inaugural pulmonary infection [43,46,51,58,72].

There is a third route by which *Aspergillus* arrives at the skin or mucosa from a neighbouring cavity, for example, paranasal or maxillary sinuses.

Cases of cheek, nose, and eyelid necrotic ulcers following rhinosinusitis have also been reported [12]. Moreover, cutaneous infections have been described in neonates, in immunosuppressed children, and after traumatic injuries, with varying treatment options [49,57,113]. For an instance, skin infections due to *A. terreus* are particularly rare [114]. Although most of these patients had leukaemia, the literature has reported other diseases, including astrocytoma [55], aplastic anaemia [46,54,55], chronic granulomatous disease [115], and agranulocytosis managed with antithymocyte globulin [50,51,61,116]. Cutaneous aspergillosis reports in immunocompromised patients are depicted in Table 5.

Aspergillosis affects 0.4–7% of hospitalized patients with burn injuries [70]. Cutaneous aspergillosis in burn patients is well described; it is not automatically related to immunosuppression [6,10,43,44]. Fungal wound infection has a high rate of mortality, especially by aspergillosis, which is around 87.1% [117]. For an instance, in an immunocompetent patient without medical history, severe fungal wound infection was related to *Aspergillus tamarii* [118].

However, all superficial and cutaneous lesions cannot be attributed to environmental *Aspergillus* that can usually colonize skin and upper respiratory tract; therefore, additional risk factors are needed to trigger an *Aspergillus* infection.

## 5. Conclusions

*Aspergillus* species are ubiquitous and saprophytic; they can cause a category of superficial and cutaneous mycoses: onychomycosis, otomycosis, and skin aspergillosis. This group of diseases caused by *Aspergillus* genus is relatively rare and poorly described.

Colonization is the initial step in aspergillosis. Fungal conidia can develop and grow on a damaged skin or after inhalation. Risk factors are various and they include: (a) environmental factors, such as climate, outdoor and hospital conditions and (b) host factors, including occupational exposure (agriculture exposure), increased local humidity and skin maceration, trauma exposure (self-induced trauma, skin injuries, burns, orthopaedic trauma), underlying medical conditions (HIV, diabetes mellitus, cancer, transplant recipients), and medical procedures (instrumentation, medical devices, catheters, bandages, drugs).

The main causative agent of otomycosis is *Aspergillus niger*. Mould onychomycosis is dominated by *Aspergillus flavus*, and cutaneous aspergillosis is caused principally by *Aspergillus fumigatus*.

Otomycosis and onychomycosis are very common diseases. Due to the incapacity of an effective keratolysis, *Aspergillus* growth needs a fragilized keratin (ablution, humidity). In this clinical group of superficial aspergilloses, immunosuppression can cause more severe and aggressive forms of otomycosis and onychomycosis.

On the other hand, the cutaneous *Aspergillus* entity is seen more in immunocompromised patients, especially after catheter insertion or medical procedure; otherwise, trauma and burn can also predispose to cutaneous *Aspergillus.*

The treatment of cutaneous and superficial aspergillosis is based on antifungal drugs (itraconazole, amphotericin B). Surgical debridement can be required, especially in the cutaneous, ulcerative, or necrotic forms (burn patients). However, aspergillosis is relatively recurrent and difficult to treat. Thus, patient education regarding predisposing factors is necessary, as highlighted in this paper.

## Figures and Tables

**Table 1:** Reports of traumatic inoculation of *Aspergillus genus.*

Traumatic Inoculation	Location	Context	Species	References
Self-induced injury	Ear	Schizophrenia	*A. flavus*	Merad et al. 2018 [7]
Burns	Skin	Burns	*A. fumigatus*	Anh-Tram et al. 2019 [6]
		Burns	*Aspergillus sp*	Schaal et al. 2015 [44]
		Thermal blast injury	*Aspergillus sp*	Klein et al. 2011 [45]
		Burns	*Aspergillus sp*	Mousssa et al. 1999 [10]
		Burns	*Aspergillus sp*	Becker et al. 1991 [42,43]
Trauma	Skin	Shaving axillae and perineum	*Aspergillus sp*	Tahir et al. 2011 [8]
		Traffic trauma	*Aspergillus sp*	Vitrat-Hinky et al. 2009 [41]
		Agricultural trauma	*A. terreus*	Ozer et al. 2000 [34]
		8 cases	*A. flavus*	Iwen et al. 1998 [5]
		4 cases	*A. fumigatus*	Iwen et al. 1998 [5]
		Trauma wound infection (HIV)	*A. glaucus*	Shetty et al. 1997 [4]
	Nail	Orthopedic trauma; Gardening	*A. flavus* *A. flavus*	Merad et al. 2019 [31] Naguchi et al. 2016 [1]
		Working in agricultural field	*A. niger*	Banu et al. 2013 [32]
		Coconut breakers	*Aspergillus sp*	Nascimento et al. 2014 [33]
Tight shoes	Nail	Tight shoes	Aspergillus	Rifai et al. 2019 [40]
Medical trauma	Ear	Non-healing surgical wound	*A. flavus*	Anderson et al. 1995 [11]
	Skin	Catheter site infection	*Aspergillus sp*	Lucas et al. 1999 [46]
				Girmenia et al. 1995 [47]
				Romero et al. 1995 [2]
				Hunt et al. 1992 [48]
				Allo et al. 1987 [49]

**Table 2:** Underlying medical conditions related to cutaneous aspergillosis.

Underlying Medical Condition	Context	Location	Species	References
HIV	HIV	Proximal nail Skin	*Aspergillus sp* *Aspergillus sp* *Aspergillus sp*	Choudhary et al. 2009 [52] Romero et al. 1995 [2] Hunt et al. 1992 [48]
Cancer	Hodgkin disease Leukemia Aplastic anemia Astrocytoma Chronic granulomatosis Leukemia	Skin Skin Skin Skin Skin Skin	*Aspergillus sp* *Aspergillus sp* *Aspergillus sp* *Aspergillus sp* *Aspergillus sp* *Aspergillus sp*	Richards et al. 2000 [57] Van Burik et al. 1998 [51] Van Burik et al. 1998 [51] Allo et al. 1987 [49] McCarty et al. 1986 [58] Carlile et al. 1978 [51]
Solid organ transplant recipients	Renal transplant	Skin	*Aspergillus sp* *Aspergillus sp*	Nampoory et al. 1996 [61] Langlois et al. 1980 [13]
	Liver transplant	Skin	*A. ustus* *A. fumigatus*	Stiller et al. 1994 [65] Pla et al. 1992 [66]
	Cardiac transplant	Skin	*Aspergillus sp*	Greenbaum et al. 1993 [67]
	Marrow transplant	Skin	*A. niger Aspergillus sp*	Johnson et al. 2009 [59] Bretagne et al. 1997 [58]
Diabetes mellitus	Diabetes mellitus	Nail	*Aspergillus sp*	Wijesuriya et al. 2015 [64]
Cytomegaloviirus Infection	Liver transplant recipient	Skin	*Aspergillus sp*	Wong et al. 2001 [68]
Liver disease	-	Skin	*Aspergillus sp*	Iwen et al. 1998 [5]

**Table 3:** Distribution of *Aspergillus* otomycosis in some countries.

Species	Country	%	References
*Aspergillus niger*	Spain India Brazil Mexico	35.9%(*n* = 390) 39.8% (*n* = 118) 20% (*n* = 103) 21% (*n* = 97)	García-Agudo et al. 2011 [87] Aneja et al. 2010 [82] Pontes et al. 2009 [86] Araiza et al. 2006 [88]
*Aspergillus flavus*	Iran India Brazil Mexico	13% (*n* = 881) 3.3% (*n* = 100) 10% (*n* = 103) 21% (*n* = 97)	Saki et al. 2013 [89] Desai et al. 2012 [90] Pontes et al. 2009 [86] Araiza et al. 2006 [88]
*Aspergillus fumigatus*	India Iran India Nigeria Brazil Ivory Cost	10% (*n* = 200) 6.2% (*n* = 881) 12.9% (*n* = 118) 5.7% (*n* = 53) 5%(*n* = 103) 4.1% (*n* = 115)	Satish et al. 2013 [91] Saki et al. 2013 [89] Aneja et al. 2010 [82] Fayemiwo et al. 2010 [92] Pontes et al. 2009 [86] Yavo et al. 2004 [93]
*Aspergillus terreus*	Irak Spain China Egypt	10.08% (*n* = 101) 1.6% (*n* = 390) 5.5% (6 cases) 3.61% (*n* = 59)	Al-Abbassi et al. 2011 [94] Garcia-Agudo et al. 2011 [87] Aneja et al. 2010 [82] Bassiouny et al. 2010 [95]
*Aspergillus nidulans*	Irak	0.84% (*n* = 101)	Al-Abbassi et al. 2011 [94]
*Aspergillus candidus*	Spain	7.1% (*n* = 390)	Garcia-Agudo et al. 2011 [87]
*Aspergillus versicolor*	China	0.87% (*n* = 115)	Jia et al. 2012 [81]

**Table 4:** *Aspergillus* onychomycosis reported in some countries.

Authors	Country	Species	Treatment	Context
Merad et al. 2020 [31]	Algeria	*A.* *flavus*	Oral terbinafin 250 mg/day + amorolfine 5% nail lacquer.	No underlying disease
Hirose et al. 2018 [104]	Japan	*A.* *subramanianii*	Terbinafine resolution after 6 month	No underlying disease
Moubasher et al. 2017 [105]	Egypt	*A. niger, A. flavus,* *A. terreus*	-	No underlying disease
Motamedi et al. 2016 [106]	Iran	*A. flavus*	-	-
Sharma et al. 2015 [107]	India	*A. tetrazonus*	-	-
Zotti et al. 2015 [108]	Italy	*A. melleus*	-	-
Wijesuriya et al. 2015 [64]	Sri Lanka	*A. niger* (76%)	-	Diabetic population
Nouripour-Sisakht et al. 2015 [109]	Iran	*Aspergillus sp*: 87.8% (135/463)	-	-
Ahmadi et al. 2012 [110]	Iran	*A. candidus*	Oral itraconazole 10 weeks (resistance to terbinafine)	No underlying disease
Zotti et al. 2010 [111]	Italy	*A. persii*	In vitro susceptibility to itraconazole	No underlying disease
Choudhary et al. 2009 [52]	India	*A. flavus*	-	HIV patient (Proximal onyxis)
Brasch et al. 2009 [112]	Germany	*A. ochraceopetaliformis*	Oral Terbinafine+ ciclopiroxolamine	No underlying disease

**Table 5:** Cutaneous aspergillosis reports in immunocompromised patients.

References	Country	Species	Context	Description
Mert et al. 2020 [61]	Turkey	*-*	Invasive aspergillosis in acute myeloid leukemia	Bullous and zosteriform lesions
Gallais et al. 2017 [72]	France	*A.* *fumigatus*	Invasive cutaneous aspergillosis in two preterm twins	Yellowish lesions on abdomen
Rogdo et al. 2014 [3]	Switzerland	*A.* *flavus*	Neonate	-
Torrelo et al. 2007 [26]	Spain	*A. flavus*	Leukemic child	Violeceous nodule of 6 cm with necrotic bullae
Lass-Florl et al. 2005 [107]	Austria	*A. terreus*	29% of cutaneous involvement in 67 invasive aspergillosis	-
Cook et al. 2003 [119]	India	*A. terreus*	Non-insulin dependent diabetes mellituswith myeloma	1 cm necrotic lesion on the right palm
Richards et al. 2000 [56]			Hodgkin’s disease	Painful, erythematous forearm nodule
Van Burik et al. 1998 [50]	USA	*A. fumigatus*	Catheter-tape-associated in HIV patient	Nodules
Shetty et al. 1997 [4]	USA	*A. glaucus*	Trauma wound associated in HIV patient	Ulcer
Smith et al. 1997 [68]	USA	*A. fumigatus*	Catheter, transparent-tape-associated in HIV patient	Pruritic, exophytic lesion
Shetty et al. 1997 [4]	USA	*A. fumigatus*	Catheter-associated in HIV patient	Nodules
Romero et al. 1995 [2]	USA	*A. fumigatus*	Catheter-associated in HIV patient	Verrucous plaque with micropustules
Girmenia et al. 1995 [46]	Italy	*A. fumigatus*	Catheter-associated in HIV patient	Indurated erythema
Iwen et al. 1993 [5]	USA	*A. fumigatus* *A. flavus*	-	-
Hunt et al. 1992 [47]	USA	*A. fumigatus*	Catheter-tape-associated in HIV patient	Umbilicated papule

## Data Availability

No new data were created or analysed in this study. Data sharing is not applicable to this article.
